# Diagnose this

**Published:** 2013

A ground-glass appearance of the cornea is noted immediately after cataract surgery (figure) and there is a +3 anterior chamber reaction. What condition do you suspect?

**Figure:**
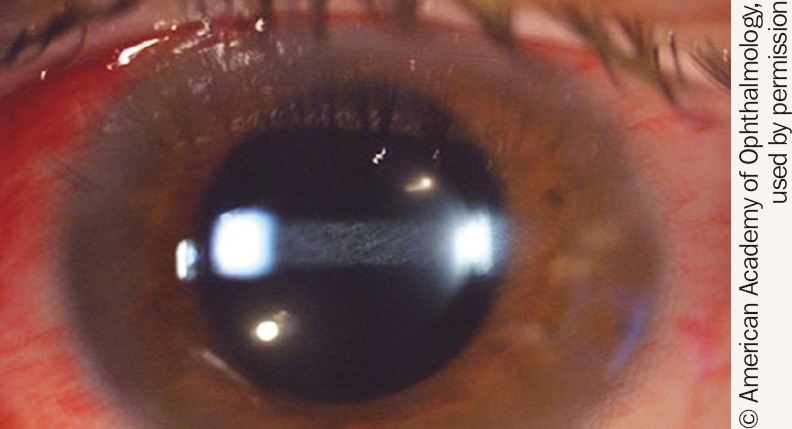


**What is the most likely diagnosis?**

- □ Endophthalmiitis
- □ Mechanical trauma to the cornea
- □ Intraoperative introduction of a toxic substance into the eye
- □ Fuchs' corneal dystrophy

## ANSWER

**Answer: Introduction of a toxic substance into the eye.** Mechanical trauma to the endothelium during surgery is considered to be the most significant factor influencing postoperative corneal oedema; however, toxic substances may inadvertently be introduced into the eye, causing immediate corneal oedema. This may come from intraocular irrigation solutions or topical and intracameral anaesthesia. Intraocular medications that have resulted in corneal toxicity include epinephrine (now available preservative free), various preparations of lidocaine, benzalkonium chloride-preserved viscoelastic, vancomycin at doses greater than 1 mg/mL, and inadvertent exposure of the endothelium to 5% povidone-iodine.

Bacterial contamination of the cleaning bath detergent for surgical instruments may also cause acute corneal oedema following cataract surgery. Oedema in these cases can be abrupt, resulting in immediate corneal swelling.

Toxic anterior segment syndrome (TASS) is an acute, sterile anterior segment inflammation following generally uneventful cataract and anterior segment surgery. Rapid onset of corneal oedema and absence of a hypopyon are the distinguishing factors in differentiating toxic corneal oedema from an infectious endophthalmitis. Most patients with TASS will develop symptoms and signs within 12 to 24 hours of the operation.

*Reproduced by kind permission of the Ophthalmic News and Education (ONE®) Network of the American Academy of Ophthalmology*. Visit **www.aao.org/one**

